# On the Reproductive Process in Plants

**Published:** 1837-10

**Authors:** 


### ON THE REPRODUCTIVE PROCESS IN PLANTS.

The author of the first article in the present volume is desirous of appending to it some views to which he has been led since that paper was written, regarding the nature of the reproductive process in the different classes of plants. The probability was there stated (p. 29,) that the *embryo* is really derived from the *pollen,* being in fact a development of one of its granules, which finds a nidus in the ovule prepared by the female system. This probability, which has now nearly attained certainty, joined to some observations recently published on the structure and development of the spores of the cryptogamia, enable us to frame a general expression of the process of reproduction in vegetables, which will be found, if we mistake not, to have an important bearing on our theory of the corresponding function in the animal kingdom.

Every vegetable, whether phanerogamous or cryptogamous, which possesses a reproductive system, forms vesicles of a peculiar character, which contain numerous minute granules moving spontaneously in the fluid of the cell. In the cryptogamia these vesicles, which are termed *spores,* when liberated from the *thecæ* in which they are developed, and placed in a situation fit for germination, undergo certain changes, presently to be described, which terminate in the formation of a new plant. In the phanerogamia, similar vesicles are formed in the *anthers,* and are called *pollen;* these, however, are incapable of producing new plants unless their development be assisted in its early stage by the parent. When the anthers rupture (and their dehiscence closely resembles that of the thecæ of cryptogamia) the pollen vesicles are conveyed upon the stigma; their outer envelope bursts, and the inner membrane is protruded in the form of long tubes which find their way down to the ovary, and there discharge into the ovule the granules contained in their fluid, one of which subsequently becomes the embryo of the new plant. Supplied by the nourishment afforded by the parent, the embryo continues its development, forming within the ovule its cotyledons, radicle, plumula, &c. and accumulating a store of nutriment for its temporary support when first called upon to maintain an independent existence. The spore of a cellular plant, on the contrary, when liberated from its parent, has to maintain an independent existence, developing its own organs and obtaining from without its supplies of nutriment. The primary changes which it undergoes are very similar to those occurring in pollen; the outer envelope is ruptured, and tubes are protruded from it; and it appears to be by the increased development of one of the contained granules that the first cell takes its origin, from which the whole plant is formed by the usual processes of growth. This first cell gives rise to others which coalesce at last into a frondose expansion, provided (in the higher orders) with radical filaments. This frondose expansion appears strictly analogous to the cotyledons of the phanerogamia; in the ferns it is merely temporary, and decays as soon as the stem and true leaves are developed; but in the lower orders it is permanent and constitutes the leaf-like portion of the general surface.

It will be seen, then, that the processes succeeding the first development of the reproductive vesicles closely correspond in all classes of plants; that in the cellulares, the embryo destined from the first to maintain an independent existence, performs a number of changes strictly analogous to those which take place in the embryo of the flowering plant whilst still supported by its parent; that the arrest of development in the former causes to assume as their permanent form that which is temporary in the embryo of the higher classes;* and (what is most important) that the essential part of the reproductive process is supplied by the male, the female only affording the conditions necessary for the increased development of the embryo by receiving it into the ovule.

*Errata in the Article referred to*:—P. 13, line 21, for *bronchial* read *branchial*. —P. 14, line 15, from bottom, for *tillænsia* read *tillandsia*.—P. 28, line 2 from bottom, for *capillary* read *carpellary*.

* The temporary formation and use of the cotyledons in the higher classes of plants contrasted with their permanency in the lower, corresponds in a very remarkable manner with the temporary appearance of *gills* (their analogous organs) in reptiles, birds, and mammalia.

